# Who Gets to Be Read? Factors Affecting Full‐Text Access Rates in Periodontology and Implantology Research

**DOI:** 10.1111/idh.70032

**Published:** 2025-12-09

**Authors:** Betina Dutra Lima, Humberto Alexander Baca Juárez, Felipe Berwaldt Islabão, Natália Marcumini Pola, Alex Nogueira Haas, Francisco Wilker Mustafa Gomes Muniz

**Affiliations:** ^1^ School of Dentistry Federal University of Pelotas Brazil; ^2^ Department of Periodontology Federal University of Pelotas Brazil; ^3^ Department of Periodontology Federal University of Rio Grande Do Sul Brazil

**Keywords:** data science, information dissemination, periodontology, publications

## Abstract

**Objective:**

This study aimed to identify the variables associated with full‐text access rates in studies published in Periodontology and Implantology.

**Methods:**

A bibliometric analysis was performed by reviewing the 30 top‐ranking journals in Dentistry, Oral Surgery and Medicine. Only journals with a number of text accesses were included. Independent variables analysed included publication date, first author's country, open access status, h‐index, study design, and thematic focus. Multivariable Poisson regression with robust variance was used.

**Results:**

A total of 3880 studies were included, revealing an access rate (number of views per month) of 83.68 ± 143.62. The journal's impact factor (rate ratio [RR]: 1.09; 95% confidence interval [95% CI]: 1.07–1.11) and being in the open access mode (RR: 1.78; 95% CI: 1.64–1.94) influenced the access rate. Moreover, studies from America (RR: 1.16; 95% CI: 1.02–1.31), Oceania (RR: 1.56; 95% CI: 1.23–1.98), and Europe (RR: 1.50; 95% CI: 1.33–1.69) had significantly higher access rates than those from Asia. Regarding study designs, randomised clinical trials (RCT) (RR: 1.70; 95% CI: 1.47–1.97) and literature reviews (non‐systematic reviews [RR: 1.65; 95% CI: 1.27–2.14] and systematic reviews [RR: 1.78; 95% CI: 1.52–2.08]) exhibited greater access than in vitro/animal studies.

**Conclusion:**

Access to articles in Periodontology is significantly linked to study design (RCTs and reviews), open access availability, and higher journal impact factors.

## 1 Introduction

The pursuit of scientific knowledge is predominantly conducted online [1], and access to full‐text articles is a critical factor influencing clinical decision‐making [2]. While numerous databases, such as PubMed, Web of Science, and Google Scholar [3], provide access to a vast array of scholarly content, significant disparities exist in the availability of full articles. These disparities can impact the dissemination of knowledge, particularly among students, clinicians, and researchers from institutions with limited access to subscription‐based journals [4].

In this sense, it is important to emphasise that the availability of full articles refers to the possibility of unrestricted or limited access to articles. In contrast, the usage of an article is measured by the rate of access to the full text and reflects the frequency with which readers view or download it. However, this relationship is not straightforward, as the effect of open access is modulated by other elements such as journal reputation and topic [5]. While availability is a relevant determinant, other factors, such as study design, journal impact factor, and thematic focus, can also significantly influence the article's usage [6].

The number of citations an article receives is traditionally regarded as a primary measure of its scientific impact. Citation analysis is widely used to evaluate the relevance and influence of research within the scientific community. However, citation metrics primarily reflect the engagement of researchers and do not necessarily capture the broader accessibility of science content. Clinicians not actively involved in research and early‐career researchers may face barriers to accessing full‐text articles, limiting their ability to fully engage with and apply the latest scientific findings [7, 8].

Restricted access to full‐text articles remains an important barrier to the dissemination of knowledge. Subscription fees, journal impact factor, and institutional policies define the availability of articles, but this is only one factor that may affect their usage rates. Understanding how these elements are associated with accessibility is essential to addressing inequalities in scientific communication [9, 10]. Therefore, this study aimed to investigate the variables associated with full‐text access rates in articles published in leading dental journals in the fields of Periodontology and Implantology.

## 2 Methods

This meta research is a bibliometric analysis of the main journals that have publications on the topics of Periodontology and Implantology. Based on the decreasing order of the impact factor indicated by the 2023 Journal of Citation Reports, the first 30 journals (considering only the first quartile of journals in this database) that presented the number of accesses to the full text were eligible. Each journal's website provides a specific tool for collecting the number of accesses (access, readers and full‐access), which indicates the number of times the full text was viewed.

### 2.1 Inclusion and Exclusion Criteria

Journals that provided any tool for evaluating the number of accesses were considered. All articles covering topics related to periodontology or implantology were included. Studies relevant to periodontology and implantology were manually identified and included according to the eligibility criteria by two trained researchers (BDL and FBI). Titles and abstracts were screened, and the basic information provided was carefully evaluated. Errata, issue summaries, and issue covers were excluded from the analysis. The inclusion period was set from 2022 (as some publishers started to make the access number tool available this year) to October 2024.

### 2.2 Data Collection

Two trained researchers (BDL and FBI) manually collected the data directly from the publishers' websites, using the access counters provided by each journal. Additionally, both researchers independently gathered data on exploratory variables. Any discrepancies were resolved through consensus between the researchers. In cases where consensus could not be reached, a third researcher (FWMGM) was consulted to solve the disagreement.

### 2.3 Outcome and Exploratory Variables

The primary outcome measured was the access rate (number of accesses per month), assessed through the access tool of each journal. Between 31 October and 2 November 2024, the access counts for all published studies were extracted from the databases. Exploratory variables included: journal (based on its impact factor), time elapsed in months between publication date and outcome collection, country of the first author (subsequently categorised as Asia, Africa, America, Europe, and Oceania), study design [categorised as randomised clinical trials (RCT), other clinical trials (CT) (case report, case series, or pilot study), non‐systematic literature review (narrative review, or clinical guidelines), systematic review, observational studies (case–control, cohort, or cross‐sectional), laboratory studies (translational research, animal model, in situ, or in vitro)], open access availability (yes or no), H‐index of the first author (dichotomised based on the median into ≤ 6 or ≥ 7), number of citations (dichotomised based on the median into ≤ 2 or ≥ 3), and theme (categorised as basic science, prevalence/incidence studies, treatment of gingivitis/periodontitis without adjuvant therapies, adjunctive therapies to treat gingivitis/periodontitis, other periodontal therapies, and peri‐implant diseases and conditions).

The H‐index data were extracted from the Web of Science database, between 21st and 28th November 2024, while the number of citations was collected from Scopus, between 3rd and 10th November 2024.

### 2.4 Statistical Analysis

Access rate (number of full‐text views per month) was defined as the main outcome. This rate was calculated as follows: number of full‐text views/[(the date that the number of accesses to full‐text was collected—date that the study was available online according to the ‘Early view’ format)/30]. The primary outcome was described using mean, standard deviation, median, and interquartile ranges. Group comparisons were conducted using either the Mann–Whitney or Kruskal‐Wallis tests. In cases where statistical significance was identified, Bonferroni corrections were applied. Afterward, for pairwise comparisons, the Mann–Whitney test was used alongside Bonferroni corrections considering multiple comparisons.

The outcome access rate presented a non‐parametric distribution (Shapiro–Wilk test presented a *p*‐value ≤ 0.001), which did not allow a linear regression analysis. Therefore, this rate was rounded to the next whole number. Furthermore, bi‐ and multivariable regressions were conducted employing Poisson regression with robust variance to estimate the rate ratio (RR) and its corresponding 95% confidence interval (95% CI). All previously mentioned exploratory variables were incorporated into the final multivariable model. Additional subgroup analyses were undertaken, distinguishing between studies with and without open access availability. For these analyses, statistical significance was established at *p* < 0.05. Multicollinearity assessments were conducted using predefined cut‐off points: variance inflation factor < 5 and tolerance > 0.2. The SPSS (version 29.0) software was used for the analyses.

## 3 Results

**FIGURE 1:**
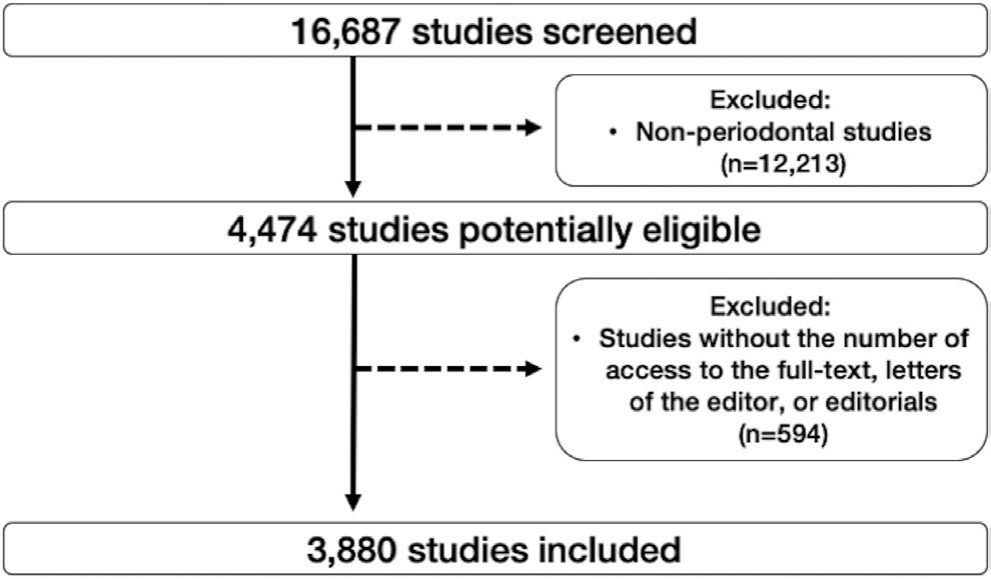
Flowchart of the studies.

The following journals were not included as the number of accesses to the full‐text was not publicly available: Journal of Prosthodontic Research and International Journal of Oral Implantology. Consequently, the 30 top‐ranking journals were screened. Among these, 16,687 articles were assessed, and 3880 were included in the final analysis. Figure 1 presents the study flowchart, while Table 1 shows the number of included studies per Journal.

**TABLE 1:** Journals retrieved for the present study and the number of included studies in each journal.

Journal	2023 Impact factor	Number of included studies	Access rate (number of access per month)
Clinical Oral Investigation	3.1	525	94.83 ± 97.43
Journal of Clinical Periodontology	5.8	369	145.57 ± 262.19
Clinical Oral Implants Research	4.8	349	98.53 ± 94.89
Journal of Prosthetic Dentistry	4.3	268	0.79 ± 0.75
Journal of Periodontal Research	3.4	265	53.05 ± 44.95
Clinical Implant Dentistry and Related Research	3.7	264	69.71 ± 60.28
Journal of Periodontology	4.2	249	92.03 ± 80.67
Journal of Dentistry	4.8	211	1.38 ± 1.56
Journal of Dental Sciences	3.4	179	0.59 ± 0.58
Oral Diseases	2.9	159	38.71 ± 38.98
Journal of Dental Research	5.7	144	73.04 ± 74.06
International Journal of Implant Dentistry	3.1	143	172.21 ± 95.14
Periodontology 2000	17.5	130	389.05 ± 259.24
Journal of Prosthodontics‐Implant Aesthetic and Reconstructive Dentistry	3.4	116	38.99 ± 35.19
Journal of Aesthetic and Restorative Dentistry	3.2	85	78.61 ± 100.70
International Dental Journal	3.2	83	1.38 ± 1.86
Frontiers in Oral Health	3.0	65	48.90 ± 23.96
Dental Materials	4.6	62	0.95 ± 0.62
International Journal of Oral Science	10.8	53	355.63 ± 309.48
Journal of the American Dental Association	3.1	33	0.58 ± 0.60
Japanese Dental Science Review	5.7	25	1.70 ± 0.88
Angle Orthodontist	3.0	19	42.64 ± 20.26
Journal of Evidence‐Based Dental Practice	4.1	18	0.88 ± 0.57
Journal of Oral Rehabilitation	3.1	17	71.06 ± 108.48
Dentomaxillofacial Radiology	2.9	17	14.21 ± 8.61
Journal of Endodontics	3.5	11	0.78 ± 0.47
International Endodontic Journal	5.4	9	112.36 ± 96.31
Progress in Orthodontics	3.5	9	240.70 ± 109.21
Oral Oncology	4.0	3	1.55 ± 1.36
Caries Research	2.9	0	—
Total		3880	

The mean number of full‐text access was 1398 ± 2767, while the access rate (number of access per month) was 83.68 ± 143.62. Further details on the univariable analysis are provided in Table S1. The results of the univariable and multivariable analyses are summarised in Table 2. Among the 3880 included articles, those with open access had significantly higher access rates (RR: 1.78; 95% CI: 1.64–1.94) compared with those without this option. Similarly, articles with a higher number of citations in Scopus exhibited a statistically significant increase in access rates (RR: 1.01; 95% CI: 1.01–1.02). In contrast, articles available online for a longer period had lower monthly access rates over time (RR: 0.96; 95% CI: 0.95–0.96).

**TABLE 2:** Bivariable and multivariable analysis of the association between exposure variables and access rate (number of access per month).

Variables	Bivariable (RR; 95% CI)	p‐value	Multivariable (RR; 95% CI)	p‐value
**Months online**	**0.97 (0.96–0.97)**	**< 0.001**	**0.96 (0.95–0.96)**	**< 0.001**
**Impact factor**	**1.14 (1.13–1.15)**	**< 0.001**	**1.09 (1.07–1.11)**	**< 0.001**
**Continent – 1st author**				
*Asia*	Ref.	**< 0.001**	Ref.	**0.023**
*America*	1.**29 (1.12–1.48)**	0.769	1.**16 (1.02–1.31)**	0.257
*Africa*	1.05 (0.75–1.49)	**< 0.001**	1.17 (0.89–1.54)	**< 0.001**
*Oceania*	**3.17 (2.22–4.53)**	**< 0.001**	**1.56 (1.23–1.98)**	**< 0.001**
*Europe*	**2.20 (1.95–2.47)**		**1.50 (1.33–1.69)**	
**Open access**				**<**
*No*	Ref.	**< 0.001**	Ref.	**0.001**
*Yes*	2.**70 (2.48–2.94)**		1.**78 (1.64–1.94)**	
**H Index— 1st author**	**1.01 (1.01–1.02)**	**< 0.001**	1.00 (0.99–1.01)	0.068
**Scopus citation**	**1.02 (1.01–1.02)**	**< 0.001**	**1.01 (1.01–1.02)**	**< 0.001**
**Study design**				
*Laboratory studies*	Ref.	**< 0.001**	Ref.	**< 0.001**
*Observational studies*	1.**31 (1.18–1.45)**	**< 0.001**	1.**25 (1.11–1.40)**	**< 0.001**
*Randomised clinical trials*	**1.77 (1.56–2.01)**	0.342	**1.70 (1.47–1.97)**	0.629
*Other clinical trials*	0.89 (0.69–1.14)	**< 0.001**	0.94 (0.74–1.20)	**< 0.001**
*Non‐systematic reviews/guideline*	**3.35 (2.80–4.01)**	**< 0.001**	**1.65 (1.27–2.14)**	**< 0.001**
*Systematic reviews*	**2.17 (1.87–2.52)**		**1.78 (1.52–2.08)**	
***Theme***				
*Basic science*	Ref.	**< 0.001**	Ref.	0.264
*Prevalence/incidence studies*	1.**30 (1.12–1.51)**	**< 0.001**	0.92 (0.79–1.07)	0.639
*Periodontal therapy (gingivitis/periodontitis)*	**1.87 (1.36–2.56)**	**0.018**	1.05 (0.85–1.30)	0.185
*Treatment of periodontitis with adjuvant therapy*	**1.27 (1.04–1.55)**	**< 0.001**	0.87 (0.72–1.07)	0.891
*Other periodontal therapies*	**1.70 (1.41–2.05)**	0.052	0.99 (0.84–1.17)	**0.003**
*Implant and peri‐implant diseases*	1.15 (0.99–1.32)		**0.81 (0.71–0.93)**	

RR: rate ratio; 95% CI: 95% confidence interval. In bold, there are significant associations (*p* < 0.05).

Regarding thematic areas, studies on implants and peri‐implant diseases had lower access rates than basic science studies (RR: 0.81; 95% CI: 0.71–0.93). However, no statistically significant differences were found among the other thematic categories.

In terms of study designs, systematic reviews (RR: 1.78; 95% CI: 1.52–2.08) and non‐systematic reviews/guidelines (RR: 1.65; 95% CI: 1.27–2.14) demonstrated higher access rates compared to laboratory studies. Randomised clinical trials also showed significantly higher access rates compared to laboratory studies (RR: 1.25; 95% CI: 1.11–1.40).

Studies authored by researchers from Europe (RR: 1.50; 95% CI: 1.33–1.69) showed significantly higher access rates compared with those authored by researchers from Asia. Similarly, studies with first authors from the Americas (RR: 1.16; 95% CI: 1.02–1.31) and Oceania (RR: 1.56; 95% CI: 1.23–1.98) also demonstrated this association.

As shown in Table S2, studies available online for more months had lower mean access, regardless of whether they were open access or not, respectively (RR: 0.96; 95% CI: 0.96–0.97; RR: 0.96; 95% CI: 0.95–0.96). The impact factor of the journals positively influenced access in both conditions, with Open Access (RR: 1.09; 95% CI: 1.07–1.11) and non‐Open Access (RR: 1.09; 95% CI: 1.07–1.12). Regarding the first author's country, only authors from Europe (RR: 1.24; 95% CI: 1.12–1.37) had greater visibility in studies without open access compared with authors from Asia. In the open‐access category, authors from America (RR: 1.34; 95% CI: 1.09–1.66), Africa (RR: 1.63; 95% CI: 1.05–2.53), Oceania (RR: 1.69; 95% CI: 1.29–2.22), and Europe (RR: 1.09; 95% CI: 1.07–1.11) had higher access than authors from Asia.

The first author's H‐index was significantly associated with higher access rates in the non–open Access category (RR: 1.01; 95% CI: 1.01–1.01). Additionally, the number of citations in Scopus was positively associated with access, whether (RR: 1.01; 95% CI: 1.01–1.02) or not (RR: 1.02; 95% CI: 1.01–1.02).

## 4 Discussion

This study aimed to evaluate the frequency of access rate and their association with various independent variables in Periodontology and Implantology research. The findings revealed that observational studies, RCTs, and guideline/literature reviews (both systematic and non‐systematic) had a higher access rate. Additionally, studies published in an open‐access format and originating from Europe also showed increased accessibility. In this context, Periodontology was highlighted as a robust field with substantial scientific evidence, being associated with high citation rates within the dental domain [11].

A bibliometric review conducted in 2020 found that the most cited articles in Periodontology 2000 were published after the year 2000 [12]. These findings support the present study, which indicates that articles available for a shorter period tend to receive more full‐text views. This trend was associated with changes in the availability of digital information [13] and the increasing production of scientific evidence in recent years [11]. Another possible explanation for the finding that articles available for a shorter period tend to receive more full‐text views is that newly published papers attract immediate attention, as readers often seek out and access recent work soon after its release. Moreover, the guideline for the treatment of stage IV periodontitis, which recorded the highest number of views (67,675 at the time of data collection), was one of the most recently published studies [14]. This aspect was associated with the observed results. Notably, additional analyses were conducted excluding this study, and a comparable trend persisted (data not shown).

The association between high‐impact factor journals and increased access rate suggests that higher accessibility is associated with greater citation frequency. When it was considered whether the study was published in open access or not, it showed higher access rates in articles with authors with higher H indices and not open access. This could suggest the influence of the H‐index in the search for studies without considering their free availability. In addition, this is also consistent with the significant association with the number of citations recorded in the Scopus database. This affiliation is related to greater visibility for a specific audience that follows journals from the study area with high‐impact factors, which are recognised worldwide as references in the field.

Analysing access rates by continent revealed that all continents, except Africa, exhibited higher rates than Asia. This disparity can be attributed to stronger economic conditions and lower inequality levels in European and Oceanic countries. One possible explanation is the greater availability of institutional and technological resources in these regions. Greater access to computers in universities, reliable internet connectivity, digital library subscriptions, and institutional support for research can facilitate readers' ability to access full‐text articles. In contrast, in contexts marked by greater inequality, structural barriers were related to limitations in the availability and use of these resources, which could help explain the differences observed. Additionally, America shows relatively higher access rates, which may have been associated with Asia's vast population and the significant barriers to providing education in rural and remote areas. These challenges hinder Asia's overall performance despite the economic strength of certain nations within the continent [15, 16, 17].

In Periodontology, observational studies, randomised clinical trials, non‐systematic reviews/guidelines, and systematic reviews had significantly higher access rates than laboratorial studies. This pattern suggests that studies with greater clinical application and that offer direct recommendations for dental practice were more frequently accessed, likely due to their immediate relevance to professionals in the field. Furthermore, other types of clinical trials, which did not follow rigorous methodological standards, did not show a significant association with access rates, indicating that their influence and visibility are limited compared with more robust clinical studies.

The significant access rates for implant and peri‐implantitis topics, when compared with basic sciences, were associated with the fact that it is an area with a smaller professional niche [18]. Also, as it is a topic with a relatively greater boom in recent years, the main source of information could be in‐person events with speakers who present new approaches, which may have reduced the search for information in studies.

Studies with a first author who has a higher H‐index tend to have more citations [19]. This may be because highly cited authors are often more influential and experienced in producing well‐structured research. However, when evaluated in a multivariable analysis, the association was not statistically significant in the present study.

The influence of the open access availability on citation rates has been previously explored and aligns with our findings [20]. Additionally, the present study found statistically significant results indicating that articles available as open access have a higher number of citations and access rates compared to those not freely accessible. This was associated with open access, facilitating the process of producing and consuming scientific content.

Globally, the open‐access availability appears to enhance access rates, reflecting a growing movement toward the democratisation of knowledge, particularly in developing countries [21]. However, in Europe, open‐access articles do not stand out as much in terms of access, as European countries have a high level of academic infrastructure and high adherence to the subscription model for scientific journals. This may have contributed to the demand for articles in both open and restricted access, considering the economic cost required to access a non‐open‐access journal.

Access to scientific articles goes beyond official platforms, with alternatives such as Sci‐Hub being widely used to circumvent financial barriers [22]. A study indicated that this platform accounts for millions of downloads annually, which may underestimate the real impact of articles when only publisher metrics are considered [23].

One limitation of this study is that data were not collected on a single day, due to the large number of studies analysed. As some indices, such as the number of hits and citations, are updated continuously, small variations may have occurred over the collection period, which may slightly impact the uniformity of the data, although the general trend of the results was most likely maintained.

According to this study, the access rate enabled the assessment of the most accessed topics for reading scientific literature for the total number of months the article was online. This may be applied to the development of science and the dissemination of scientific information, aiming to reach clinicians who will apply evidence‐based clinical practice. However, as this is an original study and included only studies from 2022, the tool is still too recent to allow for a broader evaluation. Additionally, only studies focused on Periodontology and Implantology from the same publisher with the highest impact factors were evaluated, thus limiting the extrapolation of the results. Therefore, further research is warranted, involving other dental specialties and the importance of other journals adopting the full‐text view tool to contribute to a better evaluation of science dissemination.

## 5 Conclusion

It was concluded that the higher number of accesses to Periodontology articles was significantly associated with the study design (RTC and reviews/guidelines), open‐access availability and higher journal impact factors.

## Author Contributions

B.D.L. Investigation, methodology, visualisation, and writing – original draft. F.B.I. Investigation, methodology, and writing – review and editing. H.A.B.J. Investigation, methodology, and Writing – review and editing. N.M.P. Conceptualisation, investigation, methodology, validation, and writing – review and editing. A.N.H. Methodology, validation, and writing – review and editing. F.W.M.G.M. and writing – review and editing.

## Funding

The study was partially supported by the Coordenação de Aperfeiçoamento de Pessoal de Nível Superior—Brasil (CAPES)‐Finance Code 001. All other funding was self‐supported by the authors.

## Conflicts of Interest

The authors declare no conflicts of interest.

## Supporting information


**Data S1:** Supporting Information.

## Data Availability

The data that support the findings of this study are available from the corresponding author upon reasonable request.
