# Supplementary material for: Who Gets to Be Read? Factors Affecting Full‐Text Access Rates in Periodontology and Implantology Research
Source: Int J Dent Hyg. 2025 Dec 9;24(3):362–8. doi: 10.1111/idh.70032 (PMC13309210; doi:10.1111/idh.70032)
Supplement: Supplementary file 1 — Data S1: Supporting Information. [file IDH-24-362-s001.docx]

| **Table S1. Descriptive analysis of frequencies of independent variables and their association with the number of access studies in Periodontology.** | | | |
| --- | --- | --- | --- |
| **Variables** | **N (%)** | **Access rate (number of access per month)**  **Mean±SD (Median; IQR)** | **p-value** |
| **The overall number of access (in thousand) - Mean±SD (Median; IQ)** | 3880 | 1.40±2.77 (0.72; 0.21 – 1.61) | - |
| **Access rate (number of access per month) - Mean±SD (Median; IQ)** | 3880 | 83.68±143.62 (43.07; 14.44 – 104.08) | - |
| **Months online**  *≤20 months*  *>20 months* | 1944 (50.1)  1936 (49.9) | 105.49±163.71 (62.67; 24.96 – 132.27)  61.77±116.09 (30.15; 1.68 – 73.34) | **<0.001*** |
| **Continent – 1^st^ author**  *Asia*  *America*  *Africa*  *Oceania*  *Europe* | 1557 (40.1)  793 (20.4)  82 (2.1)  43 (1.1)  1405 (36.2) | 54.99±99.72 (30.92; 1.39 – 62.85)  70.91±113.07 (36.04; 12.63 – 82.88)  57.96±89.91 (32.33; 14.45 – 67.08)  175.31±206.18 (82.06; 42.80 – 216.70)  121.38±185.18 (81.52; 29.59 – 147.42) | **<0.001&**  **<0.001* (Asia vs. America)**  0.268* (Asia vs. Africa)  **<0.001* (Asia vs. Oceania)**  **<0.001* (Asia vs. Europe)**  0.604* (America vs. Africa)  **<0.001* (America vs. Oceania)**  **<0.001* (America vs. Europe)**  **<0.001* (Africa vs. Oceania)**  **<0.001* (Africa vs. Europe)**  0.133* (Oceania vs. Europe) |
| **Open Access**  *No*  *Yes* | 1899 (48.9)  1981 (51.1) | 44.54±53.35 (32.45; 19.83 – 54.15)  121.20±186.55 (83.11; 1.55 – 156.67) | **<0.001*** |
| **H Index – 1^st^ author**  ≤6  ≥7 | 2004 (51.6)  1876 (48.4) | 69.68±106.21 (37.85; 8.90 – 89.57)  98.64±173.74 (51.59; 17.63 – 117.22) | **<0.001*** |
| **Scopus Citation**  ≤2  ≥3 | 1721 (44.4)  2159 (55.6) | 72.18±147.22 (35.34; 2.68 – 87.97)  92.85±140.03 (49.08; 19.91 – 114.13) | **<0.001*** |
| **Study design**  *Laboratory studies*  *Observational studies*  *Randomized clinical trials*  *Other clinical trials*  *Non-systematic reviews/guideline*  *Systematic reviews* | 1127 (29.0)  1354 (34.9)  392 (10.1)  174 (4.5)  475 (12.2)  358 (9.2) | 52.90±76.02 (32.41; 2.19 – 70.76)  69.41±79.33 (44.32; 15.97 –99.66)  94.12±90.94 (65.16; 33.53 – 130.87)  46.86±74.69 (22.68; 0.85 – 51.99)  178.25±317.77 (65.67; 2.70 – 209.46)  115.55±136.88 (65.92; 8.50 – 181.77) | **<0.001&**  **<0.001* (Laboratory vs. observational)**  **<0.001* (Laboratory vs. RCT)**  **0.001* (Laboratory vs. Other CT)**  **<0.001* (Laboratory vs. non-systematic review/guideline)**  **<0.001* (Laboratory vs. Systematic review)**  **<0.001* (observational vs. RCT)**  **<0.001* (observational vs. Other CT)**  **<0.001* (observational vs. non-systematic review/guideline)**  **<0.001* (observational vs. Systematic review)**  **<0.001* (RCT vs. Other CT)**  0.682* (RCT vs. non-systematic review/guideline)  0.747* (RCT vs. systematic review)  **<0.001* (Other CT vs. non-systematic review/guideline)**  **<0.001* (Other CT vs. systematic review)**  0.924* (Non-systematic review/guideline vs. systematic review) |
| ***Theme***  *Basic science*  *Prevalence/incidence studies*  *Periodontal therapy (gingivitis/periodontitis)*  *Treatment of periodontitis with adjuvant therapy*  *Other periodontal therapies*  *Implant and peri-implant diseases* | 839 (21.6)  766 (19.7)  140 (3.6)  150 (3.9)  311 (8.0)  1674 (43.1) | 68.46±105.41 (38.30; 21.02 – 77.54)  89.03±136.07 (50.94; 19.95 – 105.05)  128.32±233.04 (84.39; 19.87 – 147.09)  87.06±92.60 (44.35; 21.93 – 140.57)  116.90±163.90 (64.25; 28.68 – 140.64)  78.65±151.69 (37.66; 1.37 – 103.23) | **<0.001&**  **<0.001* (Prevalence/incidence vs. basic science)**  **<0.001* (Periodontal therapy vs. basic science)**  0.018* (Basic science vs. adjuvant treatment)  **<0.001* (Basic science vs. other therapies)**  0.309* (Basic science vs. implants)  0.021* (Prevalence/incidence vs. periodontal therapy)  0.557* (Prevalence/incidence vs. adjuvant treatment)  **0.002* (Prevalence/incidence vs. other therapies)**  **<0.001* (Prevalence/incidence vs. implant)**  0.235* (Periodontal therapy vs. adjuvant treatment)  0.843* (Periodontal therapy vs. other therapies)  **<0.001* (Periodontal treatment vs. implant)**  0.149* (Adjuvant treatment vs. other therapies)  0.006* (Adjuvant treatment vs. Implants)  **<0.001* (Other therapies vs. implants)** |
| **Subtitle:** &Kruskal-Wallis; *Mann-Whitney. P values ​​in bold signify statistical significance (p<0.05 or, when applicable, Bonferroni correction was used for multiple comparisons [significance set at 0.05/number of “pair-wise comparisons”]). | | | |

| **Table S2.** Analysis of the association between access to periodontal studies and independent variables, stratified according to whether or not there was open access. | | | | |
| --- | --- | --- | --- | --- |
| **Variables** | **No – Open access**  **(RR; 95%CI)** | **p-value** | **Yes – Open access**  **(RR; 95%CI)** | **p-value** |
| **Months online** | **0.96 (0.95 – 0.96)** | **<0.001** | **0.96 (0.96 – 0.97)** | **<0.001** |
| **Impact factor** | **1.09 (1.07 – 1.12)** | **<0.001** | **1.09 (1.07 – 1.11)** | **<0.001** |
| **Continent – 1^st^ author**  *Asia*  *America*  *Africa*  *Oceania*  *Europe* | Ref.  1.00 (0.90 -1.11)  0.84 (0.69 – 1.03)  1.21 (0.51 – 2.83)  **1.24 (1.12 – 1.37)** | 0.989  0.088  0.669  **<0.001** | Ref.  **1.34 (1.09 – 1.66)**  **1.63 (1.05 – 2.53)**  **1.69 (1.29 – 2.22)**  **1.09 (1.07 – 1.11)** | **0.007**  **0.030**  **<0.001**  **<0.001** |
| **H Index – 1^st^ author** | **1.01 (1.01 – 1.01)** | **0.016** | 1.00 (0.99 – 1.01) | 0.478 |
| **Scopus Citation** | **1.02 (1.01 – 1.02)** | **<0.001** | **1.01 (1.01 – 1.02)** | **<0.001** |
| **Study design**  *Laboratory studies*  *Observational studies*  *Randomized clinical trials*  *Other clinical trials*  *Non-systematic reviews/guideline*  *Systematic reviews* | Ref.  1.10 (0.99 – 1.23)  **1.34 (1.17 – 1.53)**  1.04 (0.84 – 1.29)  **1.21 (1.01 – 1.46)**  **1.56 (1.26 – 1.93)** | 0.069  **<0.001**  0.741  **0.043**  **<0.001** | **Ref.**  **1.34 (1.13 – 1.59)**  **1.91 (1.55 – 2.34)**  0.96 (0.69 – 1.35)  **1.92 (1.36 – 2.72)**  **1.92 (1.56 – 2.36)** | **<0.001**  **<0.001**  0.815  **<0.001**  **<0.001** |
| ***Theme***  *Basic science*  *Prevalence/incidence studies*  *Periodontal therapy (gingivitis/periodontitis)*  *Treatment of periodontitis with adjuvant therapy*  *Other periodontal therapies*  *Implant and peri-implant diseases* | Ref.  0.94 (0.82 – 1.07)  1.16 (0.93 – 1.46)  0.99 (0.80 – 1.23)  1.13 (0.94 – 1.36)  **0.82 (0.73 – 0.92)** | 0.328  0.196  0.931  0.205  **<0.001** | Ref.  0.92 (0.76 – 1.12)  1.02 (0.78 – 1.34)  0.85 (0.66 – 1.10)  0.95 (0.76 – 1.19)  **0.82 (0.68 – 0.99)** | 0.428  0.875  0.226  0.663  **0.046** |
| **Subtitle:** RR: rate ratio; 95%CI: 95% confidence interval. In bold, there are significant associations (p<0.05). | | | | |
